# Loss of Function Glucose-Dependent Insulinotropic Polypeptide Receptor Variants Are Associated With Alterations in BMI, Bone Strength and Cardiovascular Outcomes

**DOI:** 10.3389/fcell.2021.749607

**Published:** 2021-10-25

**Authors:** Hüsün Sheyma Kizilkaya, Kimmie Vestergaard Sørensen, Camilla J. Kibsgaard, Laerke Smidt Gasbjerg, Alexander S. Hauser, Alexander Hovard Sparre-Ulrich, Niels Grarup, Mette M. Rosenkilde

**Affiliations:** ^1^Department of Biomedical Sciences, Faculty of Health and Medical Sciences, University of Copenhagen, Copenhagen, Denmark; ^2^Faculty of Health and Medical Sciences, Novo Nordisk Foundation Center for Basic Metabolic Research, University of Copenhagen, Copenhagen, Denmark; ^3^Department of Drug Design and Pharmacology, University of Copenhagen, Copenhagen, Denmark; ^4^Antag Therapeutics ApS, Copenhagen, Denmark

**Keywords:** glucose-dependent insulinotropic polypeptide receptor (GIPR), single nucleotide variants (SNVs), altered receptor signaling and internalization, gut-bone axis, bone mineral density, type 2 diabetes and adiposity, blood pressure, lipids

## Abstract

Glucose-dependent insulinotropic polypeptide (GIP) and its receptor (GIPR) are involved in multiple physiological systems related to glucose metabolism, bone homeostasis and fat deposition. Recent research has surprisingly indicated that both agonists and antagonists of GIPR may be useful in the treatment of obesity and type 2 diabetes, as both result in weight loss when combined with GLP-1 receptor activation. To understand the receptor signaling related with weight loss, we examined the pharmacological properties of two rare missense *GIPR* variants, R190Q (rs139215588) and E288G (rs143430880) linked to lower body mass index (BMI) in carriers. At the molecular and cellular level, both variants displayed reduced G protein coupling, impaired arrestin recruitment and internalization, despite maintained high GIP affinity. The physiological phenotyping revealed an overall impaired bone strength, increased systolic blood pressure, altered lipid profile, altered fat distribution combined with increased body impedance in human carriers, thereby substantiating the role of GIP in these physiological processes.

## Introduction

Glucose-dependent insulinotropic polypeptide (GIP) is a gut-derived hormone that is secreted from the enteroendocrine K cells in the proximal part of the small intestinal in response to nutrient intake (Baggio and Drucker, 2007; Sonne et al., 2014). GIP, along with a related hormone, glucagon-like peptide-1 (GLP-1), constitute the incretin hormones that regulate postprandial glucose tolerance by stimulating insulin release from pancreatic β-cells (Gasbjerg et al., 2020a). In contrast to GLP-1, GIP has been demonstrated to enhance glucagon secretion in a glucose-dependent manner in healthy individuals, thus at low- and normal blood glucose levels GIP stimulates glucagon secretion from α-cells, but fails to do so at higher blood glucose levels (Christensen et al., 2011, 2015). GIP has also been ascribed a role in mediating fat deposition (Asmar et al., 2016). The GIP receptor (GIPR) belongs to the class B1 G protein-coupled receptor (GPCR) superfamily and signals through Gα_*s*_/adenylyl cyclase activation, leading to increased cyclic adenosine monophosphate (cAMP) concentrations (Holst, 2019).

The GIPR is not only expressed in pancreatic islet cells and adipocytes but has a wide expression profile including, but possibly not limited to, the heart, spleen, lung, central nervous system, and thyroid cells (Baggio and Drucker, 2007). Additionally, the GIP system is important for bone metabolism through GIPR expression on osteoblasts and osteoclasts (Bollag et al., 2000; Zhong et al., 2007; Skov-Jeppesen et al., 2021) through which GIP inhibits bone resorption as well as promotes bone formation (Tsukiyama et al., 2006; Zhong et al., 2007; Berlier et al., 2015; Skov-Jeppesen et al., 2019). Even though it is now getting recognized that GIP/GIPR is involved in bone metabolism, it is largely unknown how genetic alterations, influencing GIPR signaling, affect bone growth and resorption. The potential impact of the GIP-GIPR axis in other organ systems is similarly underinvestigated. A recent review emphasized the potential importance of GIP/GIPR in cardiovascular diseases, although details of the operation of this axis in humans are virtually unknown (Heimbürger et al., 2020).

GIP is associated with the pathophysiology of obesity and type 2 diabetes mellitus (T2D) and have therefore been the focus of therapeutic interest for many years. It is currently debated whether to use GIPR agonists or -antagonists in combination with GLP-1 agonists to treat obesity and T2D, as both combinations show promising results (Holst and Rosenkilde, 2020; Killion et al., 2020; Min et al., 2020). Clearly, there is a need to better understand the biology of the GIPR system to be able to exploit its pharmacological potential.

Genome-wide association studies (GWAS) have revealed that common variants in the *GIPR* are associated with obesity (Vogel et al., 2009; Speliotes et al., 2010) and impaired glucose- and bone mineral homeostasis (Sauber et al., 2010; Saxena et al., 2010; Torekov et al., 2014). With the exemption of rs1800437 causing the amino acid change E354Q, which leads to long-term functional impairment due to its distinct ligand binding kinetics, signaling and internalization profile (Kubota et al., 1996; Almind et al., 1998; Fortin et al., 2010; Mohammad et al., 2014; Gabe et al., 2019), the *GIPR* variants have not been functionally characterized. In a recent exome-wide association study designed to discover protein-altering variants associated with body mass index (BMI), two rare variants in *GIPR* were identified (Turcot et al., 2018). These missense variants result in amino acid changes, R190Q (rs139215588) and E288G (rs143430880). From gnomAD (Karczewski et al., 2020), the frequencies of R190Q and E288G in Europeans are 0.00093 and 0.0017, corresponding to ∼1 in 500 and ∼1 in 300 being heterozygous carriers, respectively. For each variant, heterozygote carriers of the rare allele had a ∼0.15 SD lower BMI compared to non-carriers, corresponding to an effect of ∼0.65 kg/m^2^. Interestingly, one middle-aged woman carried both rare *GIPR* mutations in heterozygote form and she weighed ∼11 kg less than the average non-carrier of the same height (Turcot et al., 2018).

Here we combine molecular pharmacological phenotyping with the physiological consequences of carrying these two rare *GIPR* variants. First, we investigated experimentally the GIP receptor binding and activation properties of the two variants, and secondly, we linked our findings to human physiology by assessing summary data of previously published studies and online portals.

## Materials and Methods

### Materials

The human GIPR that was inserted into pcDNA 3.1 plasmid (GenBank accession number: NM_000164) was synthesized and purchased from GenScript (Piscataway, NJ) along with the GIPR mutations: R190Q, E288G and the double mutant R190Q-E288G. For the real-time internalization assay, the N-terminally SNAP-tagged GIPR was synthesized and purchased from Cisbio (Codolet, France) and R190Q and E288G were introduced into the wild-type GIPR by site-directed mutagenesis according to quick-change protocol, using primers:

GCGGCCATTCTCAGCCAGGACCGTCTGC (forward for R190Q), GCAGACGGTCCTGGCTGAGAATGGCCGC (reverse for R190Q), CGCAGTGCTGGGGCCGCAACGA AGTCAAGGC (forward for E288G), GCCTTGACTTCG TTGCGGCCCCAGCACTGCG (reverse for E288G).

Human GIP(1-42) was purchased from Caslo ApS (Lyngby, Denmark). HEK293 and COS-7 cells were both purchased from ATTC (Manassas, VA). Cell medium for HEK293 was purchased from Thermo Fisher Scientific (Waltham, MA) and the cell medium for COS-7 cells were prepared in-house. Other chemicals were purchased from standard commercial sources.

### Transfection and Tissue Culture

COS-7 cells were cultured at 10% CO_2_ and 37°C in Dulbecco’s Modified Eagle Medium (DMEM) 1885 supplemented with 10% fetal bovine serum (FBS), 2 mmol/L glutamine, 180 units/mL penicillin and 45 g/mL streptomycin. HEK293 cells were cultured at 10% CO_2_ and 37°C in DMEM GlutaMAX^TM^-I supplemented with 10% FBS, 180 units/mL penicillin and 45 g/mL streptomycin. Both cell lines were transfected using the calcium phosphate precipitation method (Jensen et al., 2008) for binding and cAMP assay. For β-arrestin 2 recruitment assay, the PEI-transfection method was used and the Lipofectamine transfection method was used for the internalization assay.

Transiently transfected COS-7 cells were used in homologous competition binding assay. HEK293 cells were used in cAMP accumulation, β-arrestin 2 recruitment and internalization experiments.

### cAMP Experiments

HEK293 cells were transiently transfected with either wild-type GIPR, R190Q, E288G or the double mutation R190;E288G, and the cAMP measurements were done with an enzyme fragment complementation (EFC)-based assay (Hansen et al., 2016). In brief, the cells were seeded in white 96-well plates at a density of 35.000 per well 1 day after the transfection. The following day, the cells were washed twice with HEPES-buffered saline (HBS) and incubated with HBS and 1 mM 3-isobutyl-1-methylxanthine (IBMX) for 30 min at 37°C. The cells were then stimulated with increasing concentrations of GIP(1-42) and incubated for additional 30 min at 37°C. The HitHunterTM cAMP XS assay (DiscoverX, Herlev, Denmark) was carried out according to manufacturer’s instructions.

### Homologous Competition Binding Assay

Transiently transfected COS-7 cells expressing either wild-type GIPR, R190Q, E288G or R190Q;E288G were seeded in a clear 96-well plate 1 day after transfection. The number of cells added per well was adjusted aiming for 5–10% specific binding of ^125^I-GIP(1-42). The following day, the cells were assayed by competition binding for 3-h at 4°C using ∼15–40 pM of ^125^I-GIP(1-42) and increasing concentrations of GIP(1-42) in binding buffer (50 mmol/L HEPES buffer, pH 7.2 supplemented with 0.5% bovine serum albumin (BSA). After incubation, the cells were washed in ice-cold binding buffer and lysed with 200 mmol/L NaOH with 1% SDS for 30 min. The samples were analyzed by the Wallac Wizard 1470 Gamma Counter.

### β-Arrestin 2 Recruitment Assay

To measure β-arrestin 2 recruitment, HEK293 cells were transiently transfected with either wild-type GIPR, R190Q, E288G or R190Q;E288G and the donor Rluc8-Arrestin-3-Sp1, the acceptor mem-linker-citrine-SH3 and GPCR kinase 2 (GRK2) to facilitate β-arrestin 2 recruitment. Two days after transfection, the cells were washed with PBS and re-suspended in PBS with 5 mmol/L glucose. Subsequently, 85 μL of the cell suspension solution was transferred to its respective wells on a white 96-well isoplate followed by the addition of PBS with 5 μmol/L coelenterazine-h. After a 10 min incubation of the cells with coelenterazine-h, increasing concentration of endogenous GIP(1-42) were added and luminescence was measured by the Berthold Technologies Mithras Multilabel Reader (Rluc8 at 485 ± 40 nm and YFP at 530 ± 25 nm).

### Real-Time Internalization Assay

HEK293 parental cells transiently expressing the SNAP-tag GIPR or the variant, SNAP-tag-R190Q or—E288G were seeded in white 384-well plate after transfection, at a density of 20.000 cells per well. The following day, the medium was removed and fresh medium was added to all wells. The next day, the assay was carried out by labeling all SNAP-tagged cells with 100 nmol/L Taglite SNAP-Lumi4-Tb (donor) in OptiMEM for 60 min at 37°C. Subsequently, the cells were washed 4 × with HBBS supplemented with 1 mM CaCl_2_, 1 mM MgCl_2_, 20 mM HEPES and 0.1% BSA (internalization buffer, pH 7.4). 50 μM pre-heated fluorescein-O’-acetic acid (acceptor) was added to all wells, except wells where only donor signal was measured. The 384-plates were incubated at 37°C for 5–10 min prior to ligand addition. Then, the cells were stimulated with increasing doses of GIP(1-42), that was pre-heated at 37°C, and donor signal and internalization rate were measured every 4 min for 90 min at 37°C in PerkinElmer^TM^ Envision 2014 multi-label Reader.

### Analysis of Online High Quality Summary Statistics of R190Q and E288G

Frequencies of R190Q and E288G were from gnomAD v2.1.1 (Karczewski et al., 2020). We examined available summary data from published papers to determine the effect of *GIPR* R190Q and E288G on relevant phenotypes. Data on bone mineral density (BMD) and bone fracture risk have been contributed by Morris et al. (2019). The *p*-values P.NI and P.I were used, respectively, as recommended by the authors. The data was downloaded from http://www.gefos.org/?q=content/data-release-2018. The BMD and fracture risk summary data derive from analyses performed in UK Biobank (N_*BMD*_ = 426,824; fracture risk = 53,184 cases and 373,611 controls). Summary statistical data on body composition, obesity risk, physical activity, and cardiovascular events were derived from GeneATLAS (UK Biobank, *N* = 452,264) (Canela-Xandri et al., 2018). These summary data were downloaded from http://geneatlas.roslin.ed.ac.uk/. Summary data on circulating leptin levels (*N* = 57,232) have been contributed by Yaghootkar et al. (2020) via the NHGRI-EBI GWAS Catalog. The NHGRI-EBI GWAS Catalog is funded by NHGRI Grant Number 2U41HG007823, and delivered by collaboration between the NHGRI, EMBL-EBI and NCBI. Summary statistics were downloaded from the NHGRI-EBI GWAS Catalog (Buniello et al., 2019) for study GCST90007307 and GCST90007319 (Yaghootkar et al., 2020) on 15/12/2020 and 16/12/2020, respectively. Risk of T2D was assessed by summary statistical data (48,286 cases and 250,671 controls) contributed by Mahajan et al. (2018), and the data were downloaded from http://diagram-consortium.org/downloads.html. Results included two models either not including BMI as a covariate or adjusted for BMI (BMI adj.). The lipid levels association results were derived from summary data of an exome-wide meta-analysis (*N* = ∼350,000) contributed by Lu et al. (2017), and we downloaded the data from http://csg.sph.umich.edu/willer/public/lipids2017EastAsian/. Blood pressure and hypertension were investigated based on summary data derived from a meta-analysis of rare variants associated with blood pressure measures in European individuals (*N* = 1,164,961) performed by Surendran et al. (2020). These summary data were downloaded from https://app.box.com/s/1ev9iakptips70k8t4cm8j347if0ef2u. Data on myocardial infarction include summary statistics (*N* = 42,335 cases and 78,240 controls) contributed by the CARDIoGRAMplusC4D Consortium (Myocardial Infarction Genetics and CARDIoGRAM Exome Consortia Investigators, Stitziel et al., 2016). Data on coronary artery disease/myocardial infarction were contributed by the Myocardial Infarction Genetics and CARDIoGRAM Exome investigators and were downloaded from www.CARDIOGRAMPLUSC4D.ORG. Summary statistical data on SOFT coronary artery disease [fatal or non-fatal myocardial infarction, percutaneous transluminal coronary angioplasty or coronary artery bypass grafting, chronic ischemic heart disease, and angina; *N* = 71,602 cases and 260,875 controls (53,135 cases and 215,611 controls for the exome markers)] are derived from a meta-analysis of three GWAS, namely UK Biobank (interim release), CARDIoGRAMplusC4D 1000 Genomes-based, and the Myocardial Infarction Genetics and CARDIoGRAM Exome (Nelson et al., 2017). Data on coronary artery disease/myocardial infarction have been contributed by the CARDIoGRAMplusC4D and UK Biobank CardioMetabolic Consortium CHD working group who used the UK Biobank Resource (application number 9922). Data have been downloaded from www.CARDIOGRAMPLUSC4D.ORG. Supplementary Table 1 provides further details about the different studies and cohorts. A *p*-value below 0.05 was considered as statistically significant in analyses of specific hypotheses, while a significance threshold of 10^–4^ was applied on the phenome-wide scan in UK Biobank data.

## Results

### The Glucose-Dependent Insulinotropic Polypeptide Receptor Variants, R190Q and E288G, Show Markedly Reduced G Protein-Mediated Signaling Despite Maintained Glucose-Dependent Insulinotropic Polypeptide Binding

**FIGURE 1:**
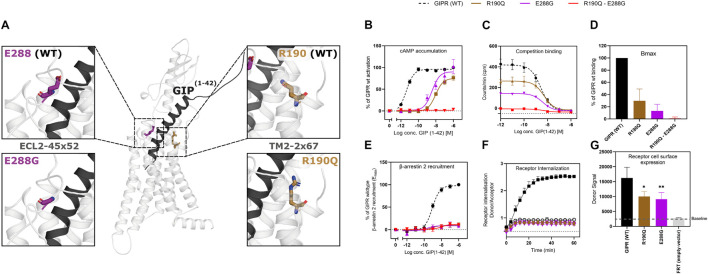
Structural localization of R190Q and E288G on the wild-type GIPR and the molecular pharmacological phenotype of the variants. **(A)** Structural illustration of the wild-type GIPR and the position of the *GIPR* variants, R190Q and E288G. **(B)** Dose-response curve in cAMP accumulation of wild-type GIPR, R190Q, E288G and double mutant. **(C)** Dose-response curves of the homologous competition binding data with [^125^I]GIP and unlabeled GIP at 4°C for 3 h of wildtype GIPR, R190Q, E288G and double mutant. **(D)** Corresponding B_*max*_ values. **(E)** Dose-response curve of β-arrestin 2 recruitment of wild-type GIPR, R190Q, E288G and double mutant. **(F)** Internalization of SNAP-GIPR, SNAP-R190Q and SNAP-E288G over time following stimulation with 1 μM GIP vs. baseline. **(G)** Receptor cell surface expression levels of SNAP-GIPR, SNAP-R190Q, SNAP-E288G, and FRT (empty-SNAP-vector). Data represent the mean ± SEM of minimum *n* = 3. Independent experiments are performed in either duplicate or triplicates. Statistical significance was assessed using an ordinary one-way ANOVA for receptor cell surface expression (*p < 0.05; **p < 0.01; as compared to wild-type response).

The residue R190 is placed in the second transmembrane (TM2) domain in position 67 of the GIPR, hence denoted R190^2.67^ (Wooten nomenclature in superscript; Wootten et al., 2013), near the first extracellular loop (ECL1), whereas E288 residue is located in the second extracellular loop (ECL2) of the receptor (Figure 1A). It has previously been shown that the N-terminal part of GIP, interacts with R190^2.67^ by forming a hydrogen bond (Smit et al., 2021; Zhao et al., 2021).

As Gα_*s*_ is the main signaling pathway for the GIPR, we assessed the impact of these two mutations either separately or in combination. This was done by measuring intracellular cAMP accumulation in transiently transfected HEK293 cells in response to increasing concentrations of GIP. Both variants displayed reduced signaling capacity compared to wild-type GIPR with a markedly decreased (>250-fold) potency of GIP with EC_50_-values of 10 nM for R190Q and 3.6 nM for E288Q, compared to the wild-type GIPR with an EC_50_-value of 4.2 pM (Table 1). R190Q reached a maximal activation (E_*max*_) of 75% of that of wild-type GIPR at 1 μM, whereas E288G reached 90%. The double mutant, R190Q-E288G resulted in a complete loss of activation through Gα_*s*_ (Figure 1B).

**TABLE 1:** Pharmacological data of *GIPR* variants, R190Q, E288G and the double mutant.

	Binding			cAMP accumulation			β -arrestin 2 recruitment		
	B_*max*_	pIC_50_	F_*mut*_	E_*max*_	pEC_50_	F_*mut*_	E_*max*_	pEC_50_	F_*mut*_

Missense variant	% of WT ± SEM	pIC_50_ ± SEM	(K_*D*_ mutation/K_*D*_ Wild-type)	% of WT ± SEM	LogEC_50_ ± SEM	(EC_50_ mutation/EC_50_ Wild-type)	% of WT ± SEM	pEC_50_ ± SEM	(EC_50_ mutation/EC_50_ Wild-type)
GIPR (WT)	100	8.6 ± 0.2		96 ± 1.4	11 ± 0.1		98 ± 2.7	9.1 ± 0.1	
R190Q	30 ± 11	8.3 ± 0.2	1.8	75 ± 2.8	8.0 ± 0.1	> 250	9.0 ± 2.5	9.1 ± 0.7	0.9
E288G	13 ± 6.1	8.4 ± 0.3	1.4	90 ± 6.2	8.4 ± 0.2	> 250	8.6 ± 2.5	9.6 ± 0.8	0.3
R190Q;E288G	0.70 ± 1.4	8.4 ± 0.5	1.5	NA	NA	NA	12 ± 2.8	8.0 ± 0.6	12.5

*All data were fitted with three-parameter logistic curve to obtain pEC_50_ and E_max_. pEC_50_ and pIC_50_ represent the negative logarithm of agonist concentration in molar that produces half the maximal response/inhibition. B_max_ is characterized as the maximum specific binding normalized to wild-type GIPR. E_max_ is characterized as the maximal response normalized to wild-type GIPR. F_mut_ is the fold change in potency, EC_50_ and in affinity, K_D mutant_, between mutants and wildtype receptor, calculated as EC_50 mutant__/_EC_50 wildtype_ and K_D mutant__/_K_D wildtype_. Data represent the mean ± SEM of at least three independent experiments performed in duplicate. NA, no activation observed.*

To determine whether the reduced cAMP formation was due to impaired agonist binding, we performed homologs competition binding, using ^125^I-GIP(1-42) as radio-ligand for the wildtype plus all three GIPR variants. Both single mutations displayed reduced binding capacity (B_*max*_) with 30% of the wild-type GIPR for R190Q, and only 13% for E288G, while the double mutant exhibited minimal binding (< 1%) (Figure 1D). The binding affinities (K_*D*_) of GIP were, however, not affected substantially as GIP bound with an affinity (K_*D*_) of 5.0 nM and 3.9 nM for R190Q and E288G, respectively, while it bound the wild-type GIPR with an affinity of 2.7 nM (Figure 1C).

### The Glucose-Dependent Insulinotropic Polypeptide Receptor Variants Display Impaired β-Arrestin 2 Recruitment and Internalization

Due to the maintained binding affinity but lower number of receptors expressed, we next set out to investigate β-arrestin 2 recruitment given its role in the desensitization and internalization of the GIPR (Gabe et al., 2018, 2020). All three variants displayed reduced ability to recruit β-arrestin 2 with an E_*max*_ of 9.0% for R190Q, 8.6% for E288G, and 12% for the double mutant compared to wild-type GIPR. There was, however, no major difference with respect to the potencies of the receptors’ ability to recruit β-arrestin 2; R190Q had an EC_50_ of 0.76 nM while E288G had an EC_50_ value of 0.23 nM compared to wild-type GIPR with an EC_50_ of 0.88 nM. The double mutant, however, displayed an EC_50_-value of 11 nM (Figure 1E). Thus, the overall maintained potency in β-arrestin 2 recruitment but lower E_*max*_ corresponded with the binding profiles of the variants.

We then performed real-time internalization experiments to determine whether the reduced β-arrestin recruitment influenced receptor internalization. Here, we used SNAP-tagged versions of the single mutant GIPR variants expressed transiently in HEK293 cells while the double mutant was omitted due to its low expression. Upon transfection with same amount of DNA of either wild-type SNAP-tagged GIPR or SNAP-tagged GIPR mutants, we observed a significantly lower receptor cell surface expression of 60% of wild-type GIPR for both single mutant variants (Figure 1G). This indicates that the reduced binding capacity of GIP to R190Q and E288G could partly be explained by the lower receptor cell surface expression. Since internalization measurements are dependent on receptor expression (Foster and Bräuner-Osborne, 2018), we next titrated receptor concentrations to obtain similar donor signal (i.e., similar cell surface expression) from the SNAP-tag in the different GIPR variants. For similar expression levels, we observed no internalization of either variant receptors (Figure 1F).

Taken together, the molecular pharmacological phenotype of the GIPR variants comprised diminished signaling through Gα_*s*_, reduced β-arrestin 2 recruitment and impaired receptor internalization. The affinity of GIP was maintained for the GIPR variants but with lower binding capacity, which could be explained by the lower receptor cell surface expression.

### The Glucose-Dependent Insulinotropic Polypeptide Receptor E288G Variant Reduces Bone Mineral Density

Since R190Q (rs139215588) and E288G (rs143430880) diminished receptor activation, we were interested in linking these functional consequences with phenotypes in humans. At first, we searched for the largest genetic studies to gather available results of the two *GIPR* variants. The present study therefore includes high quality data for R190Q and E288G from these genetic studies, in which we evaluated each *GIPR* variant separately.

**FIGURE 2:**
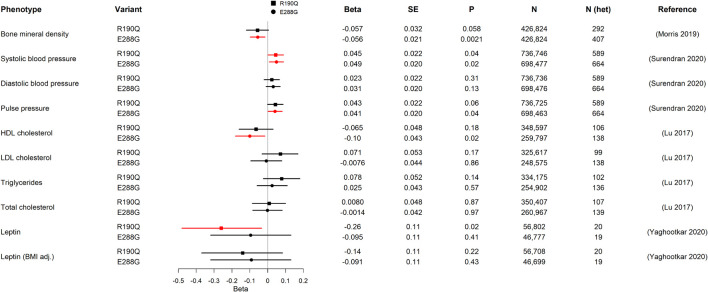
Association of *GIPR* R190Q and E288G variants with quantitative cardio-metabolic traits in GWAS. For each variant, beta, standard error (SE), the *p*-value (P), sample size (N), estimate of heterozygous variant carriers (N het), and the publication of the study from which we have gathered data from are shown. The forest plot shows the beta in SD and the 95% confidence interval. Statistically significant results are shown in red. The number of heterozygous variant carriers (N het) was estimated from allele frequency and total number of individuals (N). HDL cholesterol, high-density lipoprotein cholesterol; LDL cholesterol, low-density lipoprotein cholesterol.

We started our physiological investigation by examining bone mineral density (BMD) and fracture risk in carriers of R190Q and E288G using summary data from a study in UK Biobank with a total sample size of 426,824 individuals (Morris et al., 2019). Interestingly, E288G was associated with lower BMD (Beta –0.056 SD, *p*-value = 0.002) and R190Q showed similar effect size (–0.057 SD), but this was not statistically significant (Figure 2). None of the two *GIPR* variants seemed to be associated with an overall risk of bone fracture (Table 2).

**TABLE 2:** Association of *GIPR* variants, R190Q and E288G with relevant dichotomous phenotypes.

Trait	R190Q (rs139215588)					E288G (rs143430880)					References
	EAF	OR	95% CI	P	N	EAF	OR	95% CI	P	N	
Fracture risk	0.0014	1.001	0.80–1.25	0.99	426,795	0.0019	0.995	0.86–1.15	0.94	426,795	Morris et al., 2019
T2D	0.0015	1.19	0.84–1.69	0.56	298,957	0.0017	0.82	0.65–1.04	0.17	298,957	Mahajan et al., 2018
T2D, BMI adj.	0.0015	1.30	0.93–1.81	0.28	298,957	0.0017	0.76	0.60–0.96	0.04	298,957	Mahajan et al., 2018

	**EAF**	**Z-score**		**P**	**N**	**EAF**	**Z-score**		**P**	**N**	

Hypertension	0.0016	1.78		0.07	614,250	0.0087	1.71		0.09	548,903	Surendran et al., 2020

*EAF, effect allele frequency; SE, standard error; P, p-value; N, sample size; OR, odds ratio; BMI adj., body mass index adjusted.*

### Both Body Mass Index-Lowering Glucose-Dependent Insulinotropic Polypeptide Receptor Variants Show Effects of Cardio-Metabolic Importance

Next, we examined the association with several traits of importance for cardio-metabolic health and disease. First, we evaluated the impact of R190Q and E288G on blood pressure in summary data from a newly published paper of rare genetic variations associating with blood pressure measures, which comprised > 800,000 individuals (Surendran et al., 2020). Both *GIPR* variants were associated with higher systolic blood pressure (R190Q: 0.045 SD; E288G: 0.049 SD), although the diastolic blood pressure was not significantly different between carriers and non-carriers (Figure 2). Furthermore, the E288G variant was associated with higher pulse pressure (Figure 2), while neither of the *GIPR* variants were associated with increased risk of hypertension (Table 2).

We next examined the lipid profile to gain further insight into how R190Q and E288G with impaired GIPR signaling affected lipid homeostasis. Here we used summary statistics from an exome-chip based meta-analysis of ∼350,000 individuals (Lu et al., 2017). Carriers of R190Q did not have altered lipid levels compared to non-carriers, whereas carriers of E288G had lower high-density lipoprotein (HDL) cholesterol levels (beta = –0.10 SD, *p*-value = 0.02), yet with no changes in low-density lipoprotein (LDL), triglycerides or total cholesterol (Figure 2). Despite the impact on cardiovascular parameters, neither one of the rare *GIPR* variants, R190Q and E288G, in the present study associated with overall risk of cardiovascular events as major cause of death (Supplementary Table 2) in summary data for the UK Biobank cohort (N = 452,264) (Canela-Xandri et al., 2018).

Alterations in circulating leptin levels could be a putative mechanism of body weight regulation, and we therefore evaluated whether the two *GIPR* variants had altered levels from a genetic study of circulating leptin in early adiposity (*N* = 57,232) (Yaghootkar et al., 2020). Only R190Q was significantly associated with lowered leptin levels, although this association was lost when adjusting for BMI (Figure 2).

We also explored how the *GIPR* variants affect risk of T2D in summary data from a study of coding variants in T2D (48,286 cases and 250,671 controls) (Mahajan et al., 2018). In a model not adjusted for BMI, none of the rare *GIPR* variants were associated with risk of T2D. In contrast, a BMI-adjusted model showed that carriers of E288G had a decreased risk of T2D compared to non-carriers (OR 0.76, *p*-value = 0.04) (Table 2).

### Both Glucose-Dependent Insulinotropic Polypeptide Receptor Variants Associate With Multiple Adiposity-Related Measures

**FIGURE 3:**
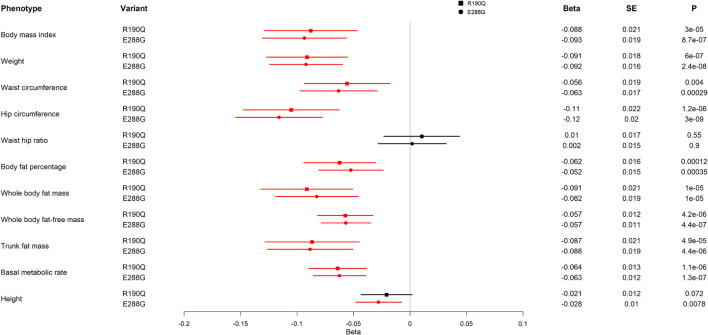
Association of *GIPR* R190Q and E288G variants with adiposity-related measurements in UK Biobank. For each variant, beta, standard error (SE), and the *p*-value (P) are shown. All results are from an analysis of rank normalized phenotypes. The forest plot shows the beta in SD and the 95% confidence interval. Statistically significant results are shown in red. The analyses include 452,264 individuals. The effect allele frequencies of GIPR R190Q and E288G are 0.001557 and 0.001915, respectively, corresponding to 352 and 433 carriers of the variants, respectively.

To further assess how the two *GIPR* variants, R190Q and E288G, impact adiposity, we evaluated adiposity-related traits using UK Biobank results from the GeneATLAS portal (*N* = 452,264) (Canela-Xandri et al., 2018). We found the same direction of association with BMI for R190Q and E288G (Figure 3), however, with a somewhat smaller effect size than previously reported (R190Q: –0.088 SD; E288G: –0.093 SD) (Turcot et al., 2018). Interestingly, carriers of either of the two *GIPR* variants had in general lower values of most adiposity-related measures compared to non-carriers; hence carriers had lower weight (R190Q: –0.091 SD; E288G: –0.092 SD), lower hip circumference (R190Q: –0.11 SD; E288G: –0.12 SD), lower waist circumference (R190Q: –0.056 SD; E288G: –0.063 SD), lower fat percentage (R190Q: –0.062 SD; E288G: –0.052 SD), lower fat mass (R190Q: –0.091 SD; E288G: –0.082 SD) and fat-free body mass (R190Q: –0.057 SD; E288G: –0.057 SD) (Figure 3). Furthermore, both variants were associated with a lower basic metabolic rate (R190Q: –0.064 SD; E288G: –0.063 SD). Despite these findings, none of the *GIPR* variant carriers significantly decreased risk of obesity (data not shown).

Finally, we investigated UK Biobank data by a phenome-wide study. Here, all above-mentioned findings at *p*-value < 10^–4^ for both *GIPR* variants were related to adiposity (Supplementary Tables 3, 4).

## Discussion

**FIGURE 4:**
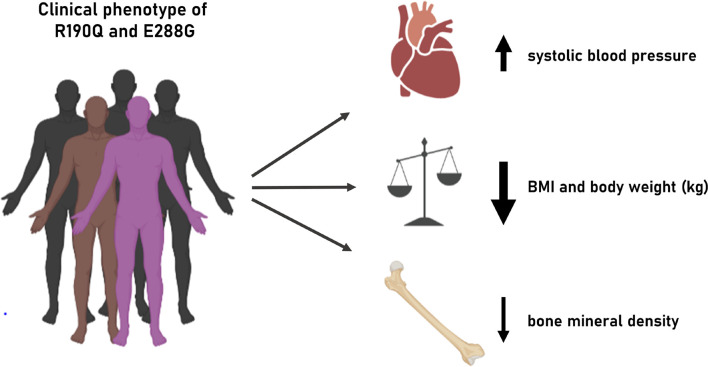
Illustration of clinical phenotype of *GIPR* R190Q and E288G. Overview of the clinical phenotypes associated with the two *GIPR* variants presented in this study.

We show that two naturally occurring rare *GIPR* variants, R190Q and E288G (rs139215588 and rs143430880, respectively), result in impaired GIPR function at the molecular level which in turn seems to impact human physiology and pathophysiology regarding adiposity, bone health and the cardiovascular system (Figure 4).

The prevailing model for ligand-binding and receptor activation of class B1 receptors, including the GIPR, is that the extracellular domain (ECD) of the receptor recognizes the C-terminal of the endogenous peptide hormone that in turn allows the N-terminal part of the ligand to position itself into the transmembrane domain (TMD) (Schwartz and Frimurer, 2017). While several structure models exist for the closely related class B1 receptors, GLP-1R and glucagon receptor (Zhang et al., 2017, 2018), the structural data of the full length human GIPR are scarce, and only few studies have been conducted to describe GIPR residues of importance for receptor activation (Yaqub et al., 2010; Cordomí et al., 2015). However, the importance of the R190- and E288 residues for GIP binding and GIPR activation was recently discussed in a study that combined MD simulations and mutagenesis experiments (Smit et al., 2021). Here, it was shown that R190 is an important residue for GIPR activation as the N-terminal part of the GIP was described to form a hydrogen bond with this residue. A similar observation was made earlier by Yaqub et al. (2010) who showed a decrease in cAMP signaling upon agonist binding. Moreover, a recent cryo-EM structure by Zhao et al. (2021) of the human GIPR in complex with GIP and a G_*s*_-heterotrimer confirmed the formation of hydrogen bond between GIP and the R190 residue. The E288 residue appears to have a bigger impact on ligand binding (5.4-fold reduction in affinity and a B_*max*_ of 32% compared to wild-type) than on activation, when substituted with an alanine (Smit et al., 2021). This is in line with the results of the present study, as we also saw a limited maximum binding capacity of 13% in E288Q, as we would expect a mutation to glycine (in E288G) to remove all functionality like alanine does (in E288A). In addition, we also observed a > 250-fold reduction in the GIP potency in G protein signaling for E288Q compared to wild-type GIPR, and supra-physiological GIP levels were needed for near maximum receptor activation. Similar impairment in terms of cAMP production was also published very recently (Akbari et al., 2021). We, in addition, found that R190Q and E288G displayed a diminished arrestin recruitment that in return resulted in a lack of receptor internalization, consistent with the previously established arrestin dependency for GIPR internalization (Gabe et al., 2018). Altogether, the functional data indicate that both *GIPR* variants disrupt the conformational changes necessary for receptor activation and arrestin recruitment, and also reduce receptor cell surface expression, while still preserving the binding of GIP.

Circulating GIP is a multi-functional incretin hormone that acts on several targets, among which bone metabolism has been the focus of several recent studies. Rodents that lack GIPR have reduced bone size, bone mass, altered bone microarchitecture- and bone turnover (Xie et al., 2005; Gaudin-Audrain et al., 2013; Mieczkowska et al., 2013). Thus, GIP analogs have been shown to improve bone composition and strength in rodents (Mabilleau et al., 2014; Vyavahare et al., 2020), while a GIPR antagonist impairs bone remodeling in humans (Gasbjerg et al., 2020b; Helsted et al., 2020). In the present study, E288G carriers had a significantly lower BMD, yet neither of the two *GIPR* variants showed a significantly increased overall bone fracture risk, possibly due to low statistical power. The common *GIPR* variant, E354Q (rs1800437), showed similar effects of lowered BMD along with increased risk of non-vertebral fractures (Torekov et al., 2014). However, E354Q shows either a similar or slightly enhanced signaling pattern as wild-type GIPR with an increased rate of receptor internalization, possibly due to a longer residence time of GIP for this mutant (Almind et al., 1998; Fortin et al., 2010; Mohammad et al., 2014; Gabe et al., 2019). As a result of decreased recycling of the receptor to the cell surface, this ultimately may result in functional impairment of the *GIPR* variant, E354Q, thus exhibiting the same phenotypic trait as R190Q and E288G.

Previous studies have already established the importance of the GIP-GIPR axis in glucose regulation. For instance, GIPR-deficient mice showed lower glucose-stimulated insulin levels and higher levels of plasma glucose (Miyawaki et al., 1999), a risk factor for T2D (Garber, 2000). In the present study, we found that E288G associated with a 24% decreased risk of T2D, whereas Turcot et al. (2018) did not detect this protective effect (Turcot et al., 2018), perhaps due to the lower sample size in the previous study [N ∼50,000 compared to ∼300,000 individuals (Table 2)]. Several GWAS have identified variants positioned in the *GIPR* locus, including the E354Q *GIPR* variant, to associate with increased 2-h glucose levels, decreased insulin secretion, insulin resistance and risk of T2D (Almind et al., 1998; Hu et al., 2010; Sauber et al., 2010; Saxena et al., 2010), further supporting the importance of the GIP-GIPR axis in glucose regulation.

Regarding the impact on the cardiovascular system, it was previously shown that GIP infusions decreased mean arterial blood pressure and increased resting heart rate (Wice et al., 2012). In fact, GIP infusions decreased diastolic blood pressure and increased heart rate during normoglycemia and hypoglycemia (Skov-Jeppesen et al., 2019; Heimbürger et al., 2020), whereas during hyperglycemia, the systolic blood pressure was increased as well (Gasbjerg et al., 2021). In our study, carriers of either *GIPR* variants had a higher systolic blood pressure and pulse pressure. Since a previous study showed no association between the two *GIPR* variants and systolic blood pressure (Turcot et al., 2018), the higher statistical power of the current study (N ∼700,000; Figure 2) compared to the study by Turcot et al. (2018) (N ∼135,000) may explain this discrepancy. Taken together, our results establish that GIPR signaling is important for the regulation of blood pressure in a manner dependent on the glycemic state.

Dysregulation of circulating lipids is also a risk factor of cardiovascular diseases. High circulating levels of GIP have shown beneficial effects on the lipid profile in humans (Møller et al., 2016), and treatment with GIPR/GLP-1R co-agonists have shown improvement of the lipid profile in patients with T2D (Frias et al., 2017, 2018). We found that carriers of E288G had significantly decreased HDL cholesterol levels without effect on other parameters of the lipid profile, suggesting that reduced GIPR signaling is involved in part of the cholesterol and lipid metabolism. These results are consistent with a previous study (Turcot et al., 2018), and the *GIPR* E354Q variant also showed a trend toward decreased HDL levels (Nitz et al., 2007). Even though carriers of R190Q and E288G have higher blood pressure and decreased HDL levels, they are not at higher risk of a cardiovascular event, and E354Q only nominally associated with cardiovascular disease (Nitz et al., 2007). Thus, reduced GIPR signaling does not seem to have fatal effects on the cardiovascular system, however, it is more likely that this study lacks statistical power to detect an effect on a clinical dichotomous phenotype even though association with a quantitative risk factor is detected. Similarly, we observe an association with BMD, yet no association with risk of fractures. Our observation that carriers of either *GIPR* variants had lower body fat mass and lean body mass than non-carriers corresponds with a previous association with lower BMI (Turcot et al., 2018), and was confirmed recently by whole-exome sequencing (Akbari et al., 2021). These results suggest that GIPR signaling contributes to regulation of body weight and body composition, and that reduced GIPR signaling is a potentially beneficial strategy against obesity. In support, obese *Gipr* knockout mice show lower body weight gain compared to wild-type mice, which may be explained by a lower fat mass, lean tissue mass and food intake, and an increased physical activity in these mice (Boer et al., 2021; Zhang et al., 2021). In the present study, we did not see an increased self-reported physical activity among carriers of R190Q or E288G. Furthermore, no increase was observed for the *GIPR* variant carriers regarding circulating leptin levels. In a previous study, obese *Gipr* knockout mice maintained leptin sensitivity compared to obese wild-type mice, and their leptin-induced anorectic effect was not inhibited by GIP infusion (Kaneko et al., 2019). If same scenario applies for humans, inadequate GIPR signaling, as for R190Q and E288G, may have beneficial effects in treatment of obesity. Further investigation in humans is needed to understand how GIPR signaling affects leptin sensitivity and long-term appetite control.

Although our results together with several studies of anti-GIPR antibodies (Gault et al., 2005; Killion et al., 2018; Min et al., 2020; Svendsen et al., 2020; Chen et al., 2021) could indicate that GIPR antagonists could protect from diet-induced obesity and improve glycemic and insulinotropic effects, other studies have shown the same for GIPR agonists (Nørregaard et al., 2018; Mroz et al., 2019; Samms et al., 2021). It is therefore still uncertain whether an agonist or an antagonist would be superior for the treatment of obesity. It is also worth noticing that the most prominent anti-obesity effect of GIPR agonists as well as antagonist is accomplished in combination with GLP-1R agonists (Killion et al., 2018, 2020; Nørregaard et al., 2018; Holst and Rosenkilde, 2020) indicating an important interplay between the two incretin hormones and their receptors.

## Conclusion

In conclusion, our results suggest that reduced GIPR signaling can have both beneficial and disadvantageous effects on human physiology. Long-term use of GIPR antagonists may be of exceptional benefit in lowering adiposity for treatment of obesity and its comorbidities, such as T2D. In contrast, long-term use of a GIPR antagonist may, to some extent, negatively affect bone metabolism and the cardiovascular system, although the effects seem to be rather small. There are various additional *GIPR* missense variants detected in the human population, which could be explored for their potential impairment and/or altered signaling properties. This may provide a more complete picture of the physiological impact of GIPR signaling and how to best exploit its therapeutic potential.

## Data Availability Statement

The datasets presented in this study can be found in online repositories. The names of the repository/repositories and accession number(s) can be found in the article/Supplementary Material.

## Author Contributions

HK, KS, MR, and NG: study design, manuscript writing—original draft. HK, MR, AS-U, and CK: functional studies. KS and NG: human genetic studies. AH: structural modeling. HK, KS, LG, AH, NG, and MR: manuscript writing—reviewing and editing. All authors revised the manuscript and approved the final version. All authors contributed to the article and approved the submitted version.

## Conflict of Interest

MR was co-founder of Antag Therapeutics and Bainan Biotech. AS-U was co-founder and CEO of Antag Therapeutics. LG was co-founder of Antag Therapeutics. The remaining authors declare that the research was conducted in the absence of any commercial or financial relationships that could be construed as a potential conflict of interest.

## Publisher’s Note

All claims expressed in this article are solely those of the authors and do not necessarily represent those of their affiliated organizations, or those of the publisher, the editors and the reviewers. Any product that may be evaluated in this article, or claim that may be made by its manufacturer, is not guaranteed or endorsed by the publisher.
