# Supplementary material for: Loss of Function Glucose-Dependent Insulinotropic Polypeptide Receptor Variants Are Associated With Alterations in BMI, Bone Strength and Cardiovascular Outcomes
Source: Front Cell Dev Biol. 2021 Oct 25;9:749607. doi: 10.3389/fcell.2021.749607 (PMC8573201; doi:10.3389/fcell.2021.749607)
Supplement: Supplementary file 1 [file Table_1.DOCX]

Supplementary Material

# Supplementary Tables:

**Supplemental Table 1. Information about cohorts and studies used in present study.**

| **Cohort/**  **study** | **Author(s)** | **Publication** | **Year** | **DOI** | **Web link publication** | **Data**  **availability** | **Study sample size** | **N cases and**  **controls** | **Trait(s)** |
| --- | --- | --- | --- | --- | --- | --- | --- | --- | --- |
| CARDIoGRAMplusC4D Consortium | Myocardial Infarction Genetics and CARDIoGRAM Exome Consortia Investigators, Stitziel NO, Stirrups KE, Masca NG, Erdmann J, et al. | Coding Variation in ANGPTL4, LPL, and SVEP1 and the Risk of Coronary Disease | 2016 | 10.1056/NEJMoa1507652 | <https://www.nejm.org/doi/10.1056/NEJMoa1507652?url_ver=Z39.88-2003&rfr_id=ori:rid:crossref.org&rfr_dat=cr_pub%20%200www.ncbi.nlm.nih.gov> | <http://www.cardiogramplusc4d.org/data-downloads/> | Up to 120,575 | 42,335 cases and 78,240 controls | Myocardial infarction |
| CARDIoGRAMplusC4D Consortium | Nelson et al. | Association analyses based on false discovery rate implicate new loci for coronary artery disease | 2017 | 10.1038/ng.3913 | <https://www.nature.com/articles/ng.3913> | <http://www.cardiogramplusc4d.org/data-downloads/> | Up to 332,477  (exome markers: up to 268,746) | 71,602  cases and 260,875 controls (53,135 cases and 215,611 controls for the exome markers) | SOFT coronary artery disease |
| Global Lipids Genetics Consortium | Lu et al. | Exome chip meta-analysis identifies novel loci and East Asian-specific coding variants that contribute to lipid levels and coronary artery disease | 2017 | 10.1038/ng.3978 | <https://www.nature.com/articles/ng.3978> | <http://csg.sph.umich.edu/willer/public/lipids2017EastAsian/> | ~ 350,000 | - | HDL cholesterol, LDL cholesterol, Triglycerides, and Total cholesterol |
| UK Biobank | Morris, Kemp, et al. | An atlas of genetic influences on osteoporosis in humans and mice | 2018 | 10.1038/s41588-018-0302-x | <https://www.nature.com/articles/s41588-018-0302-x> | <http://www.gefos.org/?q=content/data-release-2018> | Up to 426,824 | - | Bone mineral density |
| UK Biobank | Morris, Kemp, et al. | An atlas of genetic influences on osteoporosis in humans and mice | 2018 | 10.1038/s41588-018-0302-x | <https://www.nature.com/articles/s41588-018-0302-x> | <http://www.gefos.org/?q=content/data-release-2018> | Up to 426,824 | 53,184 cases and 373,611 controls | Fracture risk |
| 24 cohorts - Summary statistics of T2D associations | Mahajan et al. | Refining the accuracy of validated target identification through coding variant fine-mapping in type 2 diabetes | 2018 | 10.1038/s41588-018-0084-1 | <https://www.nature.com/articles/s41588-018-0084-1> | <http://diagram-consortium.org/index.html> | Up to 298,957 | 48,286 cases and 250,671 controls | Type 2 diabetes |
| GeneATLAS (UK Biobank) | Oriol Canela-Xandri, Konrad Rawlik & Albert Tenesa | An atlas of genetic associations in UK Biobank | 2018 | 10.1038/s41588-018-0248-z | <https://www.nature.com/articles/s41588-018-0248-z> | <http://geneatlas.roslin.ed.ac.uk/> | Up to 452,264 | - | Body fat and lean mass phenotypes, obesity, physical activity, atherosclerosis, ischemic heart disease, heart failure, and cerebrovascular diseases |
| 35 cohorts, GWAS | Yaghootkar et al. | Genetic Studies of Leptin Concentrations Implicate Leptin in the Regulation of Early Adiposity. | 2020 | 10.2337/db20-0070 | <https://diabetes.diabetesjournals.org/content/69/12/2806> | <https://www.ebi.ac.uk/gwas/publications/32917775> | Up to 57,232 | - | Circulating leptin levels |
| 75 studies from consortia (CHARGE, CHD Exome+, GoT2D:T2DGenes, ExomeBP) and UK Biobank of European ancestry | Surendran et al. | Discovery of rare variants associated with blood pressure regulation through meta-analysis of 1.3 million individuals | 2020 | 10.1038/s41588-020-00713-x | <https://www.nature.com/articles/s41588-020-00713-x#Sec17> | <https://app.box.com/s/1ev9iakptips70k8t4cm8j347if0ef2u> | Up to 1,164,961 | - | Systolic blood pressure,  Diastolic blood pressure,  Pulse pressure,  and Hypertension |

Supplemental Table 1. Here you find information about the study/cohort, the authors, publication information, web link to the publication and to the data, the maximum sample size, number of cases and controls, and which trait(s) that has been evaluated in each study/cohort.

**Supplemental Table 2. Effects of *GIPR* variants on cardiovascular events.**

|  | **R190Q (rs139215588)** | | | | **E288G (rs143430880)** | | | |  |
| --- | --- | --- | --- | --- | --- | --- | --- | --- | --- |
| **Trait** | **MAF** | **OR** | **SE (log OR)** | ***p*-value** | **MAF** | **OR** | **SE (log OR)** | ***p*-value** | **Cohort/study** |
| Myocardial infarction | 0.0017 | 0.87 | 0.19 | 0.47 | 0.0021 | 1.29 | 0.14 | 0.08 | CARDIoGRAMplusC4D Consortium |
| SOFT coronary artery disease* | 0.0014 | 0.95 | 0.16 | 0.73 | 0.0019 | 1.17 | 0.12 | 0.17 | CARDIoGRAMplusC4D Consortium |
| Atherosclerosis | 0.0016 | 0.46 | - | 0.10 | 0.0019 | 1.20 | - | 0.68 | GeneATLAS, UK Biobank |
| Ischemic heart diseases | 0.0016 | 0.97 | - | 0.71 | 0.0019 | 1.08 | - | 0.36 | GeneATLAS, UK Biobank |
| Heart failure | 0.0016 | 1.32 | - | 0.24 | 0.0019 | 0.92 | - | 0.69 | GeneATLAS, UK Biobank |
| Cerebrovascular diseases | 0.0016 | 1.03 | - | 0.88 | 0.0019 | 0.9 | - | 0.55 | GeneATLAS, UK Biobank |

Supplemental Table 2. Effect alleles of R190Q and E288G are A and G, respectively. Cardiovascular events from GeneATLAS are the ICD10 codes. *The phenotype comprises individuals having fatal or nonfatal myocardial infarction, percutaneous transluminal coronary angioplasty or coronary artery bypass grafting, chronic ischemic heart disease and angina. MAF, minor allele frequency; OR, odds ratio; SE, standard error.

**Supplemental Table 3. Phenome-wide association signals of R190Q in UK Biobank with *p*-value < 10^-4^.**

| **Trait** | **Beta** | ***p*-value** |
| --- | --- | --- |
| Leg fat mass (left) | 0.16924 | 3.1506e-07 |
| Arm fat mass (right) | 0.067011 | 4.5877e-07 |
| Leg fat mass (right) | 0.1696 | 5.4291e-07 |
| Hip circumference | 0.98718 | 8.1384e-07 |
| Arm fat mass (left) | 0.07283 | 9.2305e-07 |
| Weight | 14,367 | 9.4952e-07 |
| Whole body fat mass | 0.93895 | 1.9251e-06 |
| Arm fat percentage (right) | 0.72267 | 7.8966e-06 |
| Body mass index (BMI) | 0.44638 | 9.1488e-06 |
| Leg predicted mass (right) | 0.10191 | 1.291e-05 |
| Leg predicted mass (left) | 0.10184 | 1.4476e-05 |
| Leg fat-free mass (left) | 0.10828 | 1.5302e-05 |
| Leg fat-free mass (right) | 0.10748 | 1.6073e-05 |
| Trunk fat mass | 0.47195 | 1.6238e-05 |
| Leg fat percentage (left) | 0.47474 | 2.3515e-05 |
| Arm fat percentage (left) | 0.67512 | 3.3123e-05 |
| Basal metabolic rate | 70.578 | 3.3735e-05 |
| Whole body fat-free mass | 0.49506 | 7.668e-05 |
| Body fat percentage | 0.53016 | 8.969e-05 |
| Whole body water mass | 0.36098 | 9.134e-05 |
| Leg fat percentage (right) | 0.46109 | 9.4722e-05 |

Supplemental Table 3. The effect allele of R190Q (rs139215588) for the results in this table is G. The R190Q variant was genotyped in UK Biobank. Minor allele frequency of R190Q in UK Biobank: 0.00156. P-value for Hardy-Weinberg equilibrium: 0.51.

**Supplemental Table 4. Association signals of E288G in UK Biobank with *p*-value < 10^-4^.**

| **Trait** | **Beta** | ***p*-value** |
| --- | --- | --- |
| Hip circumference | -0.98132 | 5.4439e-08 |
| Weight | -14.327 | 7.3798e-08 |
| Leg fat-free mass (left) | -0.11133 | 8.2276e-07 |
| Leg predicted mass (left) | -0.10348 | 1.0302e-06 |
| Basal metabolic rate | -72,817 | 2.0983e-06 |
| Leg fat-free mass (right) | -0.10589 | 2.4513e-06 |
| Leg predicted mass (right) | -0.098682 | 2.8269e-06 |
| Trunk fat mass | -0.46025 | 3.1355e-06 |
| Whole body fat-free mass | -0.52171 | 3.8214e-06 |
| Whole body water mass | -0.38167 | 4.4994e-06 |
| Body mass index (BMI) | -0.41916 | 4.5566e-06 |
| Whole body fat mass | -0.7534 | 2.2762e-05 |
| Trunk fat-free mass | -0.23955 | 3.4252e-05 |
| Trunk predicted mass | -0.22734 | 4.189e-05 |
| Arm predicted mass (right) | -0.031622 | 4.2228e-05 |
| Arm fat-free mass (left) | -0.035298 | 5.2391e-05 |
| Arm fat-free mass (right) | -0.032949 | 5.7673e-05 |
| Arm predicted mass (left) | -0.032612 | 7.3867e-05 |

Supplemental Table 4. The effect allele of E288G (rs143430880) for the results in this table is G. The E288G variant was imputed in UK Biobank with an imputation score of 1. Minor allele frequency of R190Q in UK Biobank: 0.00191. P-value for Hardy-Weinberg equilibrium: 1.
